# Excision of the Larynx

**Published:** 1878-04

**Authors:** 


EXCISION OF THE LARYNX.—This formidable operation has been successfully performed in Glasgow by Dr. Foulis. Thyrotomy had been previously performed twice, and a recurrent growth removed. At length it was decided to remove the whole larynx. The patient consented, and the operation was done. The patient made a good recovery, and could dispense with artificial feeding in a fortnight. As soon as the wound contracted enough, a vulcanite tube was fitted in with a vibrating reed, which enables the patient to speak in a monotonous but loud voice.—*The Doctor.—Med. Bi-Weekly.*

